# Comparative study of children’s mental health outcomes in Tyrol, Austria, and South Tyrol, Italy, during the COVID-19 pandemic

**DOI:** 10.1007/s40211-023-00483-y

**Published:** 2023-11-21

**Authors:** Gabriele Kohlboeck, Verena Barbieri, Anna Wenter, Giuliano Piccoliori, Adolf Engl, Kathrin Sevecke, Christian J. Wiedermann, Silvia Exenberger

**Affiliations:** 1 Department of Child and Adolescent Psychiatry, Psychotherapy and Psychosomatics, Tirol Kliniken, Innsbruck, Austria; 2 Department of Child and Adolescent Psychiatry, Medical University of Innsbruck, Innsbruck, Austria; 3 Institute of General Practice and Public Health, Claudiana College of Health Professions, Bolzano, Italy; 4 Department of Psychology, University of Innsbruck, Innsbruck, Austria; 5 Department of Public Health, Medical Decision Making and Health Technology Assessment, University of Health Sciences, Medical Informatics and Technology, Hall, Tyrol, Austria

**Keywords:** Children, Mental health, COVID-19 pandemic, Tyrol, South Tyrol, Kinder, Psychische Gesundheit, COVID-19-Pandemie, Tirol, Südtirol

## Abstract

**Purpose:**

This study aimed to compare the mental health outcomes of children in North Tyrol, Austria, and South Tyrol, Italy, during the coronavirus disease 2019 (COVID-19) pandemic, considering the sociocultural and contextual differences between the two regions.

**Methods:**

The Tyrolean COVID-19 Children’s Study (TCCS: *n* = 401; June 2021 to July 2021) and the Corona and Psyche in South Tyrol 2021 Study (COP‑S; *n* = 3402; May 2021 to June 2021) were used for data analyses. Both studies employed cross-sectional designs and collected data through online questionnaires completed by children aged 7–13 years and their parents. Various psychosocial assessment tools including the Child and Adolescent Trauma Screening, Children Anxiety Test, Child Behavior Check List, Screen for Child Anxiety Related Emotional Disorders, and Health Behaviour in School-Aged Children were used in the surveys.

**Results:**

The comparison between North Tyrol and South Tyrol revealed no significant differences in perceived threats, trauma, or anxiety among children. Similarly, there were no substantial disparities in psychosomatic complaints, indicating similar manifestations of emotional distress across the two regions.

**Conclusion:**

The comparative analysis of children’s mental health outcomes in North Tyrol and South Tyrol during the COVID-19 pandemic confirmed the analogous influence of sociocultural and contextual factors on their wellbeing. Despite presumable variations in pandemic events, management strategies, and healthcare systems, the study suggests comparable resilience among children and highlights the importance of sociocultural factors in shaping their wellbeing. The findings emphasize the need for comprehensive understanding and targeted interventions to support children’s mental health during challenging times.

## Abstract (de)

**Hintergrund:**

Ziel dieser Studie war es, die psychische Gesundheit von Kindern in Tirol, Österreich, und Südtirol, Italien, während der COVID-19-Pandemie unter Berücksichtigung der soziokulturellen und kontextuellen Unterschiede zwischen den beiden Regionen zu vergleichen.

**Methoden:**

Die Tiroler COVID-19-Kinderstudie (TCCS: *n* = 401; Juni 2021 bis Juli 2021) und die Corona-und-Psyche-Südtirol-2021-Studie (COP‑S; *n* = 3402; Mai 2021 bis Juni 2021) wurden zur Datenanalyse herangezogen. Beide Studien waren als Querschnittstudien angelegt und wurden mittels Online-Fragebögen durchgeführt, die von Kindern im Alter von 7–13 Jahren und ihren Eltern ausgefüllt wurden. In den Erhebungen wurden verschiedene psychosoziale Instrumente verwendet, darunter das Child and Adolescent Trauma Screening, der Kinder-Angst-Test, die Child Behavior Check List, der Screen for Child Anxiety Related Emotional Disorders und das Health Behaviour in School-Aged Children.

**Ergebnisse:**

Der Vergleich zwischen Nordtirol und Südtirol ergab keine signifikanten Unterschiede in Bezug auf wahrgenommene Bedrohungen, Trauma und Ängste bei den Kindern. Auch bei den psychosomatischen Beschwerden gab es keine wesentlichen Unterschiede, was auf eine ähnliche Ausprägung der emotionalen Belastung in den beiden Regionen hindeutet.

**Schlussfolgerung:**

Die vergleichende Analyse der psychischen Gesundheit von Kindern in Nordtirol und Südtirol während der COVID-19-Pandemie bestätigte den analogen Einfluss soziokultureller und kontextueller Faktoren auf das Wohlbefinden der Kinder. Trotz mutmaßlicher Unterschiede bei Pandemieereignissen, Managementstrategien und Gesundheitssystemen deutet die Studie auf eine vergleichbare Widerstandsfähigkeit der Kinder hin und unterstreicht die Bedeutung soziokultureller Faktoren für das Wohlbefinden der Kinder. Die Ergebnisse betonen die Notwendigkeit eines umfassenden Verständnisses und gezielter Interventionen zur Unterstützung der psychischen Gesundheit von Kindern in herausfordernden Zeiten.

## Introduction

Globally, there has been a marked increase in mental health problems in children and adolescents as a result of the coronavirus disease 2019 (COVID-19) pandemic, and geographic variation in their incidence appears to be substantial [1]. There are a number of informative longitudinal surveys in addition to many snapshots of individual cross-sectional surveys; however, observations over longer time periods are limited and reflect heterogeneities, particularly in Europe [2]. Studies on the increased prevalence of mental and physical impairments due to the pandemic have been reported in Germany [3] and other European countries, including Norway [4], Denmark [5], the United Kingdom [6], France [7], and Switzerland [8].

During the COVID-19 pandemic, Austria and Italy exhibited notable differences in epidemiology, mortality, and management. Austria experienced fluctuating infection rates and implemented strict lockdown measures and travel restrictions. The country had a relatively low mortality rate and focused on protecting vulnerable populations and strengthening healthcare capacity. In contrast, Italy was heavily impacted in the early stages of the pandemic, with high infection and mortality rates. The country implemented nationwide lockdown measures, focused on testing and contact tracing, and worked to enhance the healthcare resources. Both countries emphasized vaccination efforts as a key strategy [9–11].

Two research teams attempted to understand the psychosocial health of children and adolescents in the Tyrol region—North Tyrol in Austria and South Tyrol in Italy. In South Tyrol, a large cross-sectional population-based study (Corona and Psyche in South Tyrol 2021 Study, short: COP‑S 2021) assessed health-related quality of life (HRQoL) and the mental health of children and adolescents in 2021, the second year of the COVID-19 pandemic [12]. At the same time, in Tyrol, a similar study on the mental health of children (Tyrolean COVID-19 Children’s Study, short: TCCS) was conducted, targeting both Tyrolean and South Tyrolean children [13, 14].

Given on the one hand the geographical proximity and historical ties between North Tyrol and South Tyrol [15], and on the other hand different healthcare and school management systems exist, are psychosomatic complaints and posttraumatic growth (PTG) comparable between North Tyrol and (South Tyrol) in TCCS? (ii) Which age- and sex-specific differences in psychosomatic complaints can be identified in children within the COP-S study in South Tyrol? Which problems had families to face daily and might there be simple solutions? (iii) Which proposals for the future can be derived from two surveys conducted partially in the same population?

Our hypothesis was that if the prevalence of mental health problems is similar in both regions despite the differences in healthcare and school management systems, we may be able to draw conclusions about the real psychosocial factors contributing to these health issues.

## Methods

### Study 1: TCCS (North Tyrol vs. South Tyrol)

#### Study design and sample

The TCCS, which was implemented in North Tyrol (Austria) and South Tyrol (Italy), aimed to investigate the impact of the COVID-19 pandemic on 3–13-year-olds, taking the perspective of children, parents, and educators into account, and was performed online using the CHES software (Evaluation Software Development, Innsbruck, Austria) between March 2020 and July 2022. Parents were recruited from schools and advertisements in local media. The eligibility criteria were living in North Tyrol or South Tyrol, children aged 3–13 years, proficiency in the German language, and the cognitive ability to fill out an online questionnaire.

For comparison with COP‑S 2021, we present child- and parent-reported responses for schoolchildren aged between 7 and 13 years at the third timepoint (June to July 2021). In this substudy, the children completed 141 online questionnaires (North Tyrol, 101; South Tyrol, 40), and the parents 402 (North Tyrol, 291; South Tyrol, 111).

The study was approved by the Ethics Committees of the Medical University of Innsbruck (no.1183/2020), and written consent was obtained from the participants’ families. The ethical vote of the Medical University of Innsbruck also applies to the German-speaking population of South Tyrol.

#### Measures

##### Child and Adolescent Trauma Screening (CATS 7–17)

Children and their parents completed the German version of the CATS for children and adolescents [16]. It gathers information about a child’s behavior, emotions, and experiences related to trauma during the last 2 weeks on a four-point symptom response scale from 0 “never,” 1 “once in a while,” 2 “half the time,” to 3 “almost always.” The CATS assesses various domains, including re-experiencing, avoidance, negative mood, and cognition, as well as arousal symptoms. The cut-off ≥ 21 of the total trauma symptom score was used as an indication of a clinically relevant level of symptoms.

##### Children Anxiety Test (KAT-III)

Perceived anxiety and trait anxiety from children’s perspectives were assessed using the German KAT-III [17]. This helps identify children who may experience elevated levels of anxiety. This test can be valuable in diagnosing anxiety disorders, tracking changes in anxiety symptoms over time, and evaluating the effectiveness of interventions aimed at reducing anxiety in children. The 18 items are rated using yes/no responses.

##### Child Behavior Checklist (CBCL)

Parents completed the German version of the CBCL for children aged 6–18 years [18]. The CBCL is designed to assess a wide range of behavioral and emotional problems in children and adolescents. The items are rated on a three-point Likert scale (0 = “absent,” 1 = “occurs sometimes,” 2 = “occurs often”). Raw scores for each scale are converted to norm-referenced T‑scores (mean [M] = 50, standard deviation [SD] = 10), with separate norms provided for each gender within the 6–11 and 12–18-year age ranges. “Clinically significant” elevations are indicated by T‑scores ≥ 64 on the broadband scales, and ≥ 70 on the syndrome scales. “Borderline” elevations range from 60–63 and 65–69 on the broadband and syndrome scales, respectively. In this study, parents were specifically asked to assess their children’s behavior in relation to internalizing problems and aggressive behavior using the CBCL. The internalizing problems scale includes items related to emotionally reactive behaviors, anxiety/depression, somatic complaints, withdrawal behavior, and sleep problems.

##### Posttraumatic growth (PTG)

PTG, defined as positive changes resulting from an individual’s struggle with traumatic or stressful events [19], was measured using the open-ended question: “What positive changes occurred for your child due to the COVID-19 crisis?” To understand the phenomenon of posttraumatic change more comprehensively, the construct was expanded to include possible negative experiences [20]. In the second open-ended question, parents were asked, “What negative changes occurred for your child due to the COVID-19 crisis?” If parents’ responses indicated one or more positive impact, PTG was scored as 1 (“yes”); if parents stated that they did not notice any positive impact, PTG was scored as 0 (“no”). The qualitative results were only quantified for “positive changes.”

#### Data analysis

Child- and parent-reported responses were compared between North and South Tyrol. For continuous variables following normal distribution, we used *t*-tests for two-group comparisons. For non-normally distributed data, Wilcoxon rank sum tests, M, and SD were used to describe continuous variables. For ordinal data, Pearson’s chi-square test and Fisher’s exact test for count data were used to compare frequencies (counts), and statistical analyses were performed using the statistical software package R Studio (MA, USA) version 2022.07.2.

The written answers of the parents (qualitative data) were analyzed with MAXQDA 2022 software (VERBI – Software, Berlin, Germany) for qualitative data. In collaboration with the second author, the last author conducted the qualitative data analysis applying the thematic analysis qualitative research method (TA; [21]). We approached TA in an inductive way, i.e., codes and themes were developed directly from the data. Our aim was to understand the possible positive and negative effects of the corona crisis on children from a parents’ perspective at this specific measurement point. With the crosstab function of MAXQDA, we compared the North Tyrolian sample with the South Tyrolian sample based on variable values.

### Study 2: COP-S 2021 study (South Tyrol)

#### Study design and sample

The questionnaire used in COP‑S 2021 was the COPSY Germany 2020 questionnaire [22]. The study design and methodology were similar to those of the Germany-wide, longitudinal, representative BELLA study [23], which is the module for examination of the mental health of children and adolescents.

The province-wide, population-based COP‑S 2021 was conducted as an anonymous online survey. In collaboration with the public schools’ administration, 38,400 families with children attending a public school were invited by email to participate. The online survey used the SoSci Survey Software, version 3.2.46 (SoSci Survey GmbH, Munich, Germany). After 1 week, the invitation was repeated. From May 28, 2021, to June 16, 2021, when schools started to open again after the lockdown, 6952 parents of children and adolescents aged 7–19 years participated in the online study. A total of 5159 questionnaires were completed. In addition, 2163 self-reports were gathered from children aged 11–19 years [12]. Of these completed and analyzed questionnaires, 3402 were from parents of children aged 7–13 years, and 939 were self-reports from children aged 11–13 years.

The study was approved by the Ethics Committees of South Tyrol (code 52-2021 on April 21, 2021). Parents consented to participate at the beginning of the online questionnaire.

#### Measures

Adolescents aged 11 to 13 years responded to the self-report version of the online survey, and parents of children aged 7–13 years answered the parent proxy version. The parent proxy survey included questions on the age and gender of children and adults, marital status, migration background, single parenthood, and parental education (CASMIN index [24, 25]).

##### Screen of Child Anxiety Disorders (SCARED)

The SCARED [25–28] is a questionnaire that contains nine items assessing recent symptoms of generalized anxiety disorder (GAD). In this study, adolescents completed the child version of the SCARED and were asked the frequency of each symptom on a three-point-scale: 0 (almost never), 1 (sometimes), 2 (often). A total score of 25 or above has been suggested to indicate the presence of clinically significant anxiety.

##### Health Behaviour in School-Aged Children (HBSC)

The HBSC [29] is a questionnaire used to assess various psychosomatic problems experienced by young people. Both parents and adolescents were asked to report the frequency of the eight symptoms that had occurred in the past week. The adolescents responded to these questions by choosing one of the following five answer categories: “rarely or never,” “about every month,” “about every week,” “more than once a week,” and “about every day.” Prevalences were calculated for all symptoms occurring at least about every month.

##### Support during the COVID-19 pandemic and qualitative data (open-ended questions)

Parents were asked where they had wished to receive support during the last months using five categories they could choose from: “dealing with school problems of my child,” “dealing with the feelings of my child,” “dealing with my child’s behavior,” “returning to normality after the lockdown for my child,” and “dealing with relations within our family.”

Furthermore, it was possible to add answers in an open field regarding other support the parents would have wished for which was not already mentioned as a category in the questionnaire.

#### Data analysis

The data were analyzed using descriptive statistics, using M and SD for metric variables, and absolute and relative frequencies for nominally and ordinally scaled variables. Chi-square tests were performed to compare categorical variables. Parents’ and children’s responses were compared using McNemar’s test. Significance levels of alpha < 0.05, < 0.01, and < 0.001 are reported. All statistical analyses were performed using SPSS version 25.0.0 (IBM Corp., Armonk, NY, USA).

## Results

### Sociodemographic factors

Tables 1 and 2 show the sociodemographic factors of the two surveys. For the TCCS study, random selection was not aimed at; thus, it is impossible to confirm that the sample comparisons are representative of the population being studied. The COP‑S 2021 data can be regarded as representative of the age and gender of the young South Tyrolean population, as well as for migration background and single parenthood of South Tyrolean families.

**Table 1:** Sociodemographic factors of the Tyrolean COVID-19 Children’s Study (TCCS)

	TCCS (June/July 2021) 7–13 years	
	North Tyrol	South Tyrol
	*N* = 290 (%)	*N* = 111 (%)
Female parent	93.8	89.2
Female child	51.2	41.4
7–10 years old	75.2	64.0
Migration background	n.a.	n.a.
Single parenthood	n.a.	n.a.
Low educational standard	n.a.	n.a.
Urban	19.7	19.1
COVID positivity within family	30.2	48.7
COVID death within family	0.3	8.1
Parental mental problems	n.a.	n.a.
Child: psychological consultation before the pandemic	5.5	1.8
Child: chronic disease before the pandemic	6.2	2.7

*n.a.* not applicable

**Table 2:** Sociodemographic factors of the Corona and Psyche in South Tyrol 2021 Study (COP-S)

	COP‑S 2021 (28.05.21–16.06.21) 7–13 years
	South Tyrol
	*N* = 3402 (%)
Female parent	88.6
Female child	49.8
7–10 years old	60.1
Migration background	11.5
Single parenthood	7.5
Low educational standard	22.9
Urban	26.4
Covid positivity within family	28.9
Covid death within family	13.5
Parental mental problems	2.8
Child: psychological consultation before the pandemic	3
Child: chronic disease before the pandemic	n.a.

*n.a.* not applicable

In both surveys, mothers responded to the survey questions in approximately 90% of parents. The proportion of girls in North Tyrol was higher (51.2%) vs. South Tyrol (41.4%) in the TCCS. Proportion of girls in COP‑S 2021 was 49.8%. In the TCCS, the proportion of 7–10 year-old children from North Tyrol was higher (75.2%) compared to South Tyrol (64%). The percentage of 7–10 year-old children in COP‑S 2021 was 60.1%. In the TCCS, the proportion of children living in a city was about the same between North Tyrol and South Tyrol (19.7% vs. 19.1%). For COP‑S 2021 this proportion was 26.4%.

The prevalence of COVID-19 was higher in the South Tyrolean subgroup of the TCCS (47.7%) than in North Tyrol (30.2%). The prevalence of COVID-19 in COP‑S 2021 was 28.9%. In TCCS, only 0.3% reported a COVID-19 death in the family in Tyrol, while the percentage was higher for TCCS respondents from South Tyrol (8.1%). The percentage reported from COP‑S 2021 was 13.5%. In TCCS, 5.5% of children from North Tyrol received psychological treatment, compared to 1.8% from South Tyrol. In COP‑S 2021, the proportion of children receiving psychological treatment was 3%.

### Parent-reported psychosomatic complaints in TCCS and COP-S 2021

Tables 3 (TCCS) and 4 (COP-S) present parent-reported psychosomatic complaints and symptoms of anxiety. The results were comparable, with no significant differences between the children from North Tyrol and South Tyrol in the TCCS. Most frequent symptoms were stubborn, sullen, or irritable (North Tyrol 47.6% vs. South Tyrol 42.9%; *p*-value 0.609); trouble sleeping (30.8% vs. 23.2%, *p*-value 0.150); nervous, highly strung, or tense (29.4% vs. 29.5%; *p*-value 0.934).

**Table 3:** Parent-reported psychosomatic complaints for the Tyrolean COVID-19 Children’s Study (TCCS)

TCCS (June/July 2021) 7–13 years			
CBCL	North Tyrol % [95% CI]	South Tyrol % [95% CI]	*p*-value
Headache	24.2 [19.3; 29.5]	24.1 [16.7; 33.4]	0.749
Stomachache	27.3 [22.2; 32.8]	25.9 [18.3; 35.3]	0.822
Unhappy, sad, depressed	29.4 [24.1; 34.9]	26.8 [19.0; 36.3]	0.205
Stubborn, sullen, or irritable	47.6 [41.7; 53.5]	42.9 [33.9; 53.0]	0.609
Nervous, highly strung, or tense	29.4 [24.1; 34.9]	29.5 [21.4; 39.2]	0.934
Trouble sleeping	30.8 [25.4; 36.4]	23.2 [15.9; 32.4]	0.150
Feels dizzy or lightheaded	11.8 [8.3; 16.0]	5.4 [2.0; 16.11]	0.060

**Table 4:** Parent reported psychosomatic complaints for the Corona and Psyche in South Tyrol 2021 Study (COP‑S)

COP‑S 2021 (28.05.21–16.06.21) 7–13 years	
HBSC	% [95% CI]
Headache	23.8 [22.3; 25.4]
Stomachache	26.6 [25.0; 28.3]
Backache	8.5 [7.5; 9.6]
Feeling low	28.7 [27.0; 30.4]
Irritability/bad temper	63.3 [61.5; 65.1]
Feeling nervous	36.1 [34.3; 37.9]
Difficulties in getting to sleep	35.2 [33.4; 36.9]
Feeling dizzy	6.9 [6.0; 7.9]

### Self-reported psychosomatic complaints in TCCS and COP-S 2021

The results for self-reported psychosomatic complaints are presented in Tables 5 (TCCS) and 6 (COP-S). In the TCCS, similar rates of psychosomatic complaints could be found between children in North Tyrol and in South Tyrol. Most frequent symptoms were sleeping problems (57.4% North Tyrol vs. 65% South Tyrol), anger fits (59.4% vs. 47.5%), and not being able to have good or happy feelings (28.7% vs. 37.5%).

**Table 5:** Self-reported psychosomatic complaints in Tyrolean COVID-19 Children’s Study (TCCS)

TCCS (June/July 2021) 7–13 years			
Child reports	*N* = 101	*N* = 40	
CATS, KAT-III	North Tyrol % [95% CI]	South Tyrol % [95% CI]	*p*-value
CATS: Strong feelings in your body when you are reminded of what happened (sweating, heart beating fast, upset stomach)	18.8 [11.7;27.8]	27.5 [14.6; 43.9]	0.256
CATS: Not being able to have good or happy feelings	28.7 [20.2;38.6]	37.5 [22.7; 54.2]	0.310
CATS: Feeling mad. Having fits of anger and taking it out on others	59.4 [49.2;69.1]	47.5 [31.5; 63.9]	0.199
KAT-III: I am nervous	26.7 [18.4;36.5]	32.5 [18.6; 49.1]	0.494
CATS: Any trouble falling or staying asleep in the last 2 weeks	57.4 [47.2;67.2]	65.0 [48.3; 79.4]	0.409
KAT-III: I am anxious	24.8 [16.7;34.3]	25.0 [12.7; 41.2]	0.976

**Table 6:** Self-reported psychosomatic complaints in Corona and Psyche in South Tyrol 2021 Study (COP-S)

COP‑S 2021 (28.05.21–16.06.21) 11–13 years	
	*N* = 939
HBSC	South Tyrol % [95% CI]
Headache	32.5 [29.3; 35.8]
Stomach-ache	30 [26.9; 33.2]
Backache	22.2 [19.4; 25.1]
Feeling low	33.2 [30.0; 36.5]
Irritability/bad temper	58.2 [54.8; 61.6]
Feeling nervous	39.9 [36.6; 43.3]
Difficulties in getting to sleep	40.6 [37.5; 44.1]
Feeling dizzy	15.1 [12.7; 17.7]
Symptoms of anxiety	22.8 [20.0; 25.8]

In COP‑S 2021 (Table 6), children reported irritability/bad temper (58.2%), difficulties getting sleep (40.6%), and nervousness (39.9%) as the most frequent symptoms.

### Psychosomatic complaints by gender and age in COP-S 2021

**Fig. 1:**
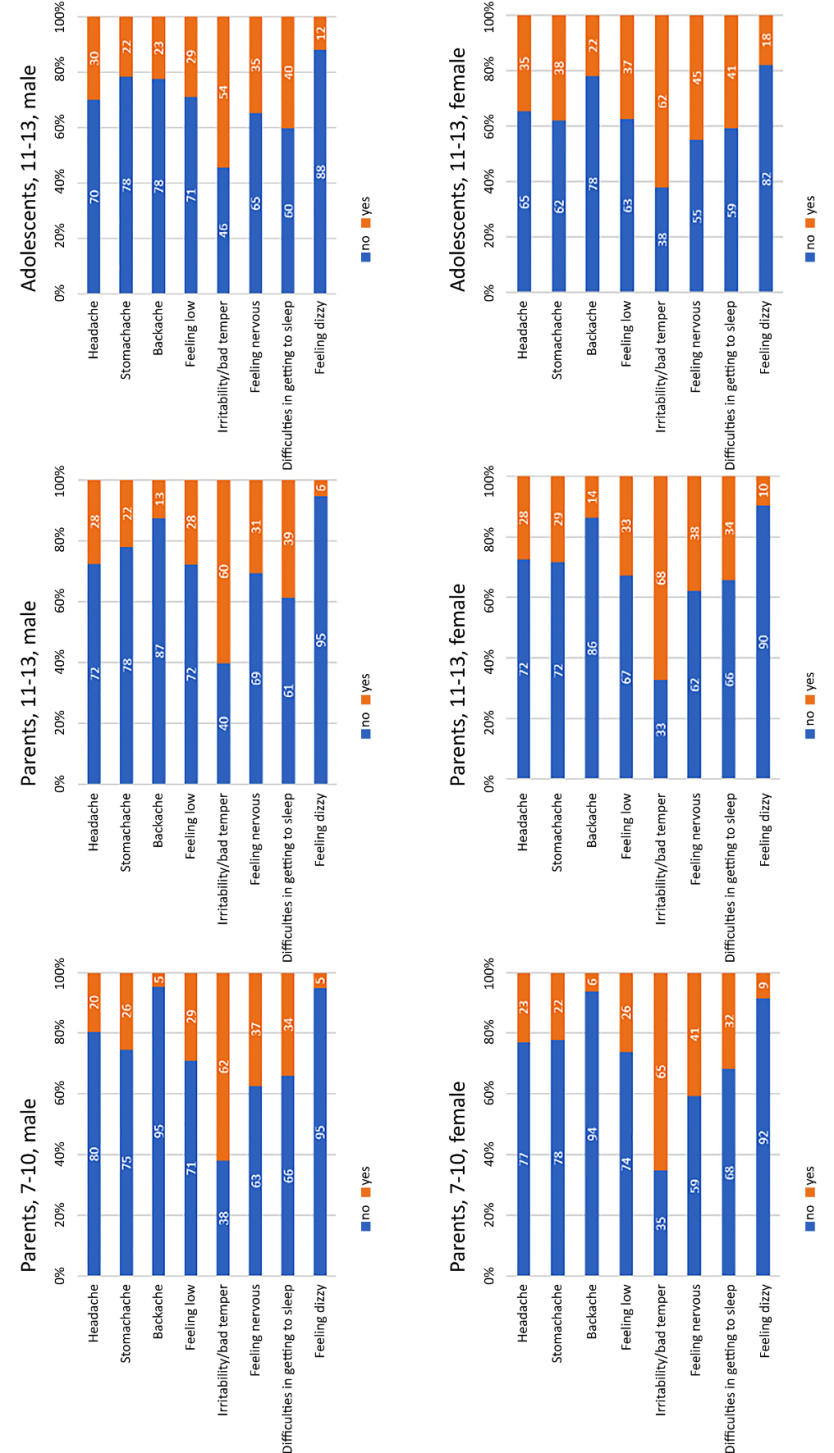
Psychosomatic complaints at least once a week per gender and age group in Corona and Psyche in South Tyrol 2021 Study (COP-S)

Age- and sex-specific psychosomatic complaints in the COP‑S 2021 survey are shown for the age groups 7–10 years and 11–13 years in Fig. 1. Significant differences between boys and girls were found in parents’ reports of children aged 7–10 years (*p* = 0.005), with girls feeling more frequently nervous (40.6%) than boys (37.3%; and for 11–13-year-olds [*p* < 0.014]) and with girls having more frequent stomachaches (28.5% vs. boys 22.2%). Significant differences between boys and girls could also be found in self-reports (11–13 years). Girls reported more frequent symptoms for stomachache (38.1% vs. boys 21.6%; [*p* < 0.001]), feeling low (girls 37.4% vs. boys 28.8%; [*p* = 0.008]), irritability, bad temper (girls 62.1% vs. boys 54.3%; [*p* = 0.023]), feeling nervous (girls 45% vs. boys 34.7%; [*p* = 0.002]), and feeling dizzy (girls 18% vs. boys 12%; [*p* = 0.015]). Significant differences in boys’ parents’ reports between age groups were found, with 11–13 year-old boys having more frequent headaches (27.7% vs. 7–10 year-old boys, 20%; *p* < 0.001), backache (12.7% vs. 4.6%; *p* < 0.001), and dizziness (6.5% vs. 3.3%; *p* = 0.018). Significant differences regarding boys between parents and children’s answers were found for backache, with boys reporting more frequent backache (22.5% vs. 12.7%; *p* < 0.001) and sleeping difficulties (40.2% vs. 38.8%). Parents reported more frequent irritability/bad temper (60.4% vs. 54.3%; *p* < 0.001). Significant differences between parent reports and girls’ self-reports were shown for headaches (girls 34.8% vs. parents 27.6%; *p* = 0.001), stomachache (38.1% vs. 28.5%; *p* < 0.001), backache (21.9% vs. 13.8%; *p* < 0.001), feeling nervous (45% vs. 37.8%), sleeping difficulties (40.9% vs. 34.4%), and dizziness (18% vs. 9.6%). Parents reported more frequent irritability/bad temper (68%) than girls (62.1%; *p* < 0.001).

### Child-reported threat, exposure, trauma, and anxiety assessed in TCCS

Table 7 presents the mean values (M) and standard deviations (SD) of child-reported threats, exposure, trauma, and anxiety in the TCCS, comparing North Tyrol and South Tyrol. No significant differences were observed in any of the scales. In North Tyrol and South Tyrol, 28.6% and 24.1% of the children met the criterion for clinically significant internalizing problems, respectively. However, when examining social problems, children from North Tyrol exhibited significantly higher social problems than children from South Tyrol. Apart from this finding, no other significant differences in problem behavior or emotional problems were observed between the children in the two regions (Table 8).

**Table 7:** Differences in threat, exposure, trauma, and anxiety between Tyrol and South Tyrol in the Tyrolean COVID-19 Children’s Study (TCCS): child reports

	North Tyrol *N* = 101	South Tyrol *N* = 40	Test
Score	Mean (SD) N %	Mean (SD) N %	Wilcoxon rank-sum test, Chi^2^-Test
Threat total score	0.44 (0.4)	0.36 (0.4)	*p* = 0.2048
Exposure total score (weighted)	0.11 (0.2)	0.15 (0.2)	*p* = 0.2254
CATS total trauma symptom score	13.1 (11.8)	11.8 (9.6)	*p* = 0.8296
Cut-off score greater 21 = probable PTSD	22 (21.8%)	10 (25%)	*P* = 0.6215
CATS re-experiencing score	3.2 (3.5)	3.0 (3.1)	*p* = 0.5905
CATS avoidance score	1.2 (1.6)	1.2 (1.5)	*p* = 0.5536
CATS negative mood/cognitions score	4.0 (4.2)	3.9 (3.6)	*p* = 0.6714
CATS arousal score	4.7 (3.8)	3.7 (2.9)	*p* = 0.2359
KAT-III total anxiety score	2.2 (3.2)	2.0 (2.7)	*p* = 0.7495
KAT-III worry score	1.3 (1.8)	1.2 (1.6)	*p* = 0.9636
KAT-III anxiety score	0.6 (0.9)	0.5 (0.9)	*p* = 0.7811
KAT-III helplessness score	0.3 (0.7)	0.2 (0.3)	*p* = 0.2643

**Table 8:** Child Behavior Check List (CBCL) Parent’s Report in the Tyrolean COVID-19 Children’s Study (TCCS)

		North Tyrol	South Tyrol	Wilcoxon rank-sum test
Anxious/depressed (T-score)	Mean (std)	59.8 (10.3)	58.3 (9.1)	*p* value: 0.3324
Withdrawn/depressed (T-score)	Mean (std)	57.7 (9.8)	56.9 (9.1)	*p* value: 0.6521
Somatic complaints (T-score)	Mean (std)	56.8 (8.5)	55.2 (7.2)	*p* value: 0.1195
Social problems (T-score)	Mean (std)	63.8 (18.5)	59.3 (15.4)	*p* value: 0.0250
Thought problems (T-score)	Mean (std)	58.3 (11.6)	57.3 (11.0)	*p* value: 0.4207
Attention problems (T-score)	Mean (std)	60.6 (11.0)	58.6 (9.6)	*p* value: 0.1539
Aggressive behavior (T-score)	Mean (std)	59.4 (10.6)	58.1 (9.3)	*p* value: 0.3439
Internalizing (T-score)	Mean (std)	56.5 (12.6)	54.6 (11.9)	*p* value: 0.2499

**Table 9:** Posttraumatic Growth (PTG) and posttraumatic depreciation (PTD) between Tyrol and South Tyrol in Tyrolean COVID-19 Children’s Study (TCCS), open questions parent reports

	North Tyrol	South Tyrol		North Tyrol	South Tyrol
–	–	–	*Loss of light-heartedness*	3.9%	5.9%
*PTG—family strength, spirituality (see personal strength, spirituality)*		–	*PTD—weakening of the own person*	–	–
Strength, spirituality	4.0%	3.5%	–	–	–
–	–	–	Insecurity	1.1%	0.0%
*PTG values & virtues (see appreciation of life)*	–	–	*PTD—little appreciation of life*	–	–
Outdoor activities, nature (+)	2.0%	0.0%	–	–	–
More appreciation, virtues, doing good for others (+)	11.3%	10.5%	Values, virtues	1.1%	1.5%
Mindfulness, deceleration, dealing with time	9.3%	15.8%	Listlessness, little motivation, boredom	7.8%	11.7%
*PTG—new competences and experiences (see new possibilities)*		–	*PTD—few new opportunities*	–	–
Autonomy	14.0%	12.3%	–	–	–
Homeschooling, kindergarten	4.7%	3.5%	Homeschooling, quality of school	27.8%	14.7%
Digitalization	4.7%	8.8%	Digitalization—media consumption	10.0%	11.8%
–	–	–	Fears of the future, uncertainty, change	8.9%	8.8%
–	–	–	Little or no input	1.1%	0.0%
*PTG—importance of intra- and extra-familial relationships (see relationships with others)*			*PTD-weakened relationships with others*	–	–
Dad & sibling relationship (+)	8.7%	15.8%	–	–	–
Lots of time with parents/family	28.0%	19.3%	Withdrawal, self-doubt	6.7%	11.8%
Extended relationships	1.3%	1.8%	Loss of contact, left alone, lonely	11.1%	16.2%
Growing together as a family, cohesion, joint actions (+)	12.0%	8.8%	Weakening of the family	11.1%	14.7%
–	–	–	Loss of social competence, splitting, stigmatization	9.4%	2.9%
%	100%	100%	–	100%	100%
*N* = documents	372 (72.2%)	143 (27.8%)	–	372 (72.2%)	143 (27.8%)

### Qualitative analysis of open questions in TCCS

The results of the qualitative analysis (Table 9) showed that the identified themes were consistent with the original PTG dimensions described by Tedeschi and Calhoun [19]. Only the dimension “spiritual and existential change” could not be identified as a separate growth and depreciation dimension. The following themes were extracted: “family strength, spirituality” (characterized by a growing together of the family), “values and virtues” (basic human values such as benevolence came to the fore), “new competences and experiences” (changes in the children’s development and experience), and “importance of intra- and extra-familial relationships” (qualitative as well as quantitative aspects of relationships were of great meaning). Parallel to the themes with the PTG dimensions, themes consistent with posttraumatic depreciation (PTD) were found, namely “weakening of the own person,” “little appreciation of life,” “few new opportunities,” and “weakened relationships with others.” “Weakening of the own person” was mentioned only by North Tyroleans, all other topics were mentioned by both groups; however, the number of statements varied between the two groups. It is clear that the most significant event during the corona crisis was the lockdown with accompanying quarantine in March 2020, as even in June and July 2021, parents related to this point in time.

### Qualitative analysis of open questions in COP-S 2021

Parents were asked where they had wished to receive support during the last months using five categories: 51.5% selected “dealing with school problems of my child,” 44.5% “dealing with the feelings of my child,” 30.5% “dealing with my child’s behavior,” 18% “returning to normality after the lockdown for my child,” and 14.7% “dealing with relations within our family.”

Furthermore, it was possible to add answers in an open field, i.e., the parents could fill out other support they would have wished for which was not already stated as a category in the questionnaire. The most frequently mentioned problem was the combination of homeschooling and smart work. Parents proposed opening schools even during the lockdown. In addition, many parents mentioned the need for more personal contact between teachers and parents during home schooling, as well as more support regarding peer problems between children. Moreover, reduced social contacts and, consequently, an extended use of digital media was perceived as a big problem: parents proposed to return to normality as soon as possible and to get support on how to teach meaningful use of digital media to their children. Finally, access to physiotherapy and psychotherapies was reduced during the pandemic, and families often had to deal with problems alone. Parents proposed extending public resources.

## Discussion

We investigated mental health outcomes from two online surveys (TCCS in North Tyrol and South Tyrol, and COP‑S 2021 in South Tyrol) during the COVID-19 pandemic. Despite differences in pandemic events, management, and healthcare systems [9–11], we found no significant differences in perceived threats, exposure, trauma, anxiety, or psychosomatic complaints between North Tyrol and South Tyrol in the TCCS study. As we did not analyze the existing differences in the healthcare and school systems according to the prevalence of mental health outcomes, our findings might be based on a hypothetical basis, and other unknown factors might also have led to our results. However, these findings suggest that children have a potential resilience and highlight the importance of sociocultural factors in shaping their wellbeing and their mental health during challenging times.

### Sociodemographic factors

The TCCS study in South Tyrol had a slightly lower proportion of girls compared to North Tyrol, and also a lower percentage of 7–10-year-old children than North Tyrol. These differences may be attributed to recruitment methods. South Tyrol experienced higher COVID-19 death rates than North Tyrol [30, 31], and fewer South Tyrolean children received psychological treatment than North Tyrolean children. Differences in available mental health programs may contribute to this variation [32–34].

### Psychosomatic symptoms

Both regions (North Tyrol and South Tyrol) reported similar rates of psychosomatic complaints. In the COPSY-Germany study [3], psychosomatic complaints increased during the pandemic in children and adolescents aged 11–17 years. The comparative study showed comparable rates of headaches, stomachaches, feeling low, and feeling dizzy in the TCCS between North and South Tyrol. Significant sex and age group differences were found in parent-reported psychosomatic complaints in the COP‑S 2021. Approximately 30% of children experienced at least one psychosomatic symptom, with prevalence rates varying based on methodological differences and other factors. According to a meta-analysis [35], prepandemic prevalence rates ranged from 5 to 83%, with a pooled prevalence of 31.0%.

### Psychosocial wellbeing and qualitative responses

Regarding children’s perceived threat, exposure, trauma, behavior, and emotional problems, no significant differences were found between North Tyrol and South Tyrol in the TCCS, except for higher social problems reported by children from North Tyrol, possibly due to stricter quarantine measures. There were no significant differences in PTG and PTD themes between the two regions, except for “weakening of the own person” found only in North Tyroleans. While no comparative survey results between the TCCS and COP‑S 2021 can be presented, qualitative analysis of responses from the COP‑S 2021 study highlighted common challenges faced by parents, including dealing with school problems, handling their child’s feelings and behavior, remote schooling challenges, reduced social contacts, increased digital media use, and limited access to therapies.

### Anxiety and trauma

The KAT-III and SCARED are used to assess anxiety in children. The TCCS found no significant differences in trauma between North Tyrolean (21.8%) and South Tyrolean children (25%). Traumatic events may cause short-term distress, but in complex family situations, symptoms can persist and lead to psychiatric disorders [36]. All symptoms, including psychosomatic complaints and anxiety, should be considered as warning signs in children and adolescents to prevent serious psychopathology beyond the pandemic.

### Practical implications and future directions

Based on the findings of these two surveys in the same population, future proposals include the need for ongoing monitoring of children’s psychological health during crises like the COVID-19 pandemic, using regular surveys for valuable data, and targeted interventions. Addressing psychosomatic symptoms is crucial, as they can provide valuable indications of potential mental health problems, reflecting underlying emotional or psychological distress. Comprehensive support mechanisms should be implemented in healthcare and school systems [13]. Specialized trauma experts may promote children’s PTG [14] and deliver mental health care in nontraditional settings, such as schools and primary care, which may be especially effective [37, 38]. Collaborative research between neighboring regions can offer insights into the factors influencing children’s mental health and inform evidence-based policies and interventions. The impact of differences in the education and healthcare systems on psychological complaints warrants further exploration.

### Limitations

Comparative studies on children’s psychosocial wellbeing in the neighboring North Tyrol and South Tyrol regions offer valuable insights, but there are limitations to consider. Both studies relied on self-reported data from parents and children and were potentially affected by bias. Conducted at a specific point in time, they provide a snapshot of children’s wellbeing, and longitudinal studies can enhance understanding over time. Focusing on a specific geographical area, they may not fully represent the diverse experiences of larger populations. Confounding factors such as socioeconomic status have not been extensively explored. Despite these limitations, these studies have provided valuable information on children’s psychological health during the COVID-19 pandemic.

## Conclusion

The results showed comparable symptom patterns, supporting the hypothesis that pandemic and school system differences between North Tyrol and South Tyrol may not substantially affect children’s mental health. The similar prevalence of psychosomatic complaints and anxiety suggests the contribution of other psychosocial factors. Therefore, we suggest that microsystem factors (parent–child relationships) and mesosystem factors (school and relationships to peers) are more important than macrosystem disparities. This analysis provides valuable insights into effective interventions and mental health monitoring. Addressing psychosomatic complaints and providing comprehensive support to the healthcare system are essential. Collaborative research and data sharing between regions can further promote children’s wellbeing. These studies lay the groundwork for future research and intervention in this region.
